# *In vitro* activity of contezolid against gram-positive bacteria isolated from pediatric patients with invasive infections

**DOI:** 10.1128/spectrum.02949-25

**Published:** 2026-09-23

**Authors:** Xinrong Li, Rubo Li, Suyun Qian, Quan Wang

**Affiliations:** 1 Pediatric Intensive Care Unit, Beijing Children's Hospital, Capital Medical University, National Center for Children's Health 12517, Beijing, China; Quest Diagnostics Nichols Institute, Chantilly, Virginia, USA

**Keywords:** contezolid, linezolid, *Staphylococcus aureus*, *Streptococcus pneumoniae*, pediatric, antimicrobial resistance

## Abstract

Contezolid, a novel oxazolidinone, exhibits potent activity against multidrug-resistant gram-positive pathogens, but data in pediatric populations remain limited. This study evaluated the *in vitro* activity of contezolid against *Streptococcus pneumoniae* (*n* = 53) and *Staphylococcus aureus* (*n* = 92) isolates recovered from sterile-site specimens of pediatric patients with invasive infections (2018–2023). MICs were determined by broth microdilution. For *S. pneumoniae*, contezolid MICs ranged from 0.125 to 1.000 µg/mL (MIC_50/90_, 0.250/0.500 µg/mL), with comparable activity against penicillin-susceptible and penicillin-nonsusceptible isolates. Contezolid exhibited low MIC values with a relatively narrow distribution across serotypes and specimen sources, without marked differences. For *S. aureus*, contezolid MICs ranged from 0.250 to 2.000 µg/mL (MIC_50/90_, 0.500/1.000 µg/mL), with equivalent activity against methicillin-susceptible *Staphylococcus aureu*s (MSSA) and methicillin-resistant *Staphylococcus aureus* (MRSA). All isolates were susceptible, with most MICs ≤ 0.500 µg/mL and a minority (*n* = 25) at 1–2 µg/mL, mostly involving isolates from blood. Contezolid exhibited potent and consistent *in vitro* activity against gram-positive pathogens isolated from invasive infections in pediatric patients, including MRSA and PNSP, with stable susceptibility profiles across major clonal lineages. These findings demonstrate favorable *in vitro* activity of contezolid against clinically significant isolates.

**IMPORTANCE:**

This study provides an evaluation of the *in vitro* activity of contezolid against gram-positive bacterial isolates obtained exclusively from pediatric patients with invasive infections. By characterizing susceptibility patterns across resistance phenotypes and molecular backgrounds, our findings offer important baseline data to inform future clinical studies of contezolid in pediatric populations.

## INTRODUCTION

Invasive infections caused by gram-positive bacteria continue to pose a significant clinical and public health challenge due to their associated morbidity and severity, particularly in vulnerable populations, such as young children. *Staphylococcus aureus* (*S. aureus*) and *Streptococcus pneumoniae* (*S. pneumoniae*) remain predominant causes of both community- and hospital-acquired invasive infections worldwide (1, 2). The widespread and prolonged use of antimicrobial agents has contributed to a steady increase in antimicrobial resistance (AMR) among these organisms (3). Linezolid and vancomycin have been regarded as cornerstone therapies for infections caused by multidrug-resistant (MDR) gram-positive bacteria. However, vancomycin resistance in *Enterococcus* spp. has become widespread, and increasing vancomycin MICs (vancomycin creep) have been reported in *S. aureus*, both of which may compromise therapeutic outcomes (4, 5). In addition, cases of linezolid-resistant *S. aureus* have also been reported (6, 7).

Contezolid, a novel oxazolidinone, has emerged as a promising candidate for treating MDR gram-positive infections, with demonstrated potent *in vitro* activity against a broad spectrum of clinically relevant organisms, including methicillin-resistant *Staphylococcus aureus* (MRSA), penicillin-non-susceptible *Streptococcus pneumoniae* (PNSP), coagulase-negative staphylococci (CoNS), *Enterococcus* spp., and various *Streptococcus* spp. (8, 9). Compared with linezolid, contezolid exhibits markedly reduced risk of bone marrow suppression and monoamine oxidase inhibition, suggesting a more favorable safety profile (10). Additionally, several studies have shown that contezolid exerts robust *in vitro* activity against MDR gram-positive bacteria in mixed-age populations (9). However, most *in vitro* studies to date have focused on isolates from adult patients, and data on pediatric isolates, especially those from invasive infections, remain limited. Considering the differences in antimicrobial resistance patterns, molecular epidemiology, and virulence characteristics of *S. pneumoniae* and *S. aureus* isolates between pediatric and adult populations, this study was designed to assess the *in vitro* activity of contezolid against isolates recovered from pediatric invasive infections (11, 12). The findings are expected to inform the potential clinical utility of contezolid for treating invasive gram-positive infections in children.

## MATERIALS AND METHODS

### Bacterial isolates

A total of 145 non-duplicate clinical isolates were obtained from pediatric patients (aged ≤18 years) with invasive infections at Beijing Children’s Hospital between January 2018 and December 2023, including 53 *S. pneumoniae* and 92 *S. aureus* isolates. Invasive infections were defined based on clinical diagnosis supported by microbiological and laboratory evidence. In most cases, pathogens were recovered from normally sterile body sites in the presence of compatible clinical manifestations (13). Only one isolate per patient was included. Species identification and routine antimicrobial susceptibility testing were performed by the Clinical Microbiology Laboratory of Beijing Children’s Hospital as part of standard diagnostic procedures. Invasive isolates were selected because they are often associated with more severe clinical presentations and may differ from non-invasive isolates in antimicrobial resistance profiles and population structure, thereby providing a stringent context for evaluating *in vitro* antimicrobial activity (14).

Penicillin MICs for *S. pneumoniae* were determined using the Phoenix 100 Automated Microbiology System (BD, Sparks, MD, USA) as part of routine antimicrobial susceptibility testing. Penicillin susceptibility of *S. pneumoniae* was interpreted according to Clinical and Laboratory Standards Institut (CLSI) M100, 35th (15). Meningitis breakpoints were applied to cerebrospinal fluid (CSF) isolates (susceptible ≤0.06 µg/mL; resistant ≥0.12 µg/mL), whereas oral penicillin V breakpoints were applied to non-CSF isolates (susceptible ≤0.06 µg/mL; intermediate 0.12–1 µg/mL; resistant ≥2 µg/mL). For epidemiological analyses, isolates with penicillin MIC ≤ 0.06 µg/mL were classified as penicillin-susceptible *S. pneumoniae* (PSSP), and those with MIC ≥0.12 µg/mL were classified as PNSP, as oral penicillin V breakpoints provide a more stringent classification than parenteral non-meningitis breakpoints (16).

Methicillin resistance in *S. aureus* was initially determined using the cefoxitin disk diffusion method. According to CLSI M100 (15), isolates with cefoxitin inhibition zone diameters ≤ 21 mm were classified as MRSA, whereas those with diameters ≥ 22 mm were classified as methicillin-susceptible *Staphylococcus aureu*s (MSSA). To further confirm methicillin resistance, the presence of the *mecA* gene was detected by polymerase chain reaction (PCR). The cefoxitin disk diffusion and *mecA* PCR results were fully concordant, with all cefoxitin-resistant isolates testing positive for *mecA* and all cefoxitin-susceptible isolates testing negative for *mecA*.

MLST typing and serotyping were performed to assess whether the *in vitro* activity of contezolid varies across different genetic backgrounds. Antimicrobial resistance in *S. pneumoniae* and *S. aureus* has been shown to be closely associated with specific serotypes and clonal lineages, with certain high-risk clones exhibiting significantly elevated resistance rates (17–19). Serogroups of *S. pneumoniae* were determined by the Quellung reaction using the Pneumotest-Latex kit (Statens Serum Institute, Copenhagen, Denmark), with serotypes further identified using specific factor antisera (20). Multilocus sequence typing (MLST) of *S. aureus* was performed by PCR amplification of seven housekeeping genes, and sequence types (STs) were assigned using the MLST database (17).

### Antimicrobial susceptibility testing

All isolates were previously stored at −70°C and subcultured twice on tryptic soy agar supplemented with 5% sheep blood (blood agar; Oxoid Ltd., UK) prior to testing to ensure viability and purity. Standard powders of contezolid and linezolid were provided by Shanghai MicuRx Pharmaceutical Co., Ltd. Antimicrobial susceptibility testing was performed using the reference broth microdilution method according to CLSI standard M07. Cation-adjusted Müeller–Hinton broth (CAMHB; Oxoid Ltd., UK) was used for antimicrobial susceptibility testing of *S. aureus*, and CAMHB supplemented with 5% lysed horse blood was used for *S. pneumoniae* (21). Broth microdilution panels were prepared on the day of testing and used immediately without prior freezing. Twofold dilutions of contezolid and linezolid from 0.125 to 4 µg/mL were tested. To minimize inter-batch variability, panel preparation and testing were performed in a single experimental run using the same batches of media and antimicrobial solutions. Since CLSI breakpoints for contezolid are not yet available, susceptibility was interpreted according to the MIC criteria in the official Chinese prescribing information: susceptible at MIC ≤ 2 µg/mL and resistant at MIC ≥ 4 µg/mL for *S. pneumoniae*; and susceptible at MIC ≤ 4 µg/mL and resistant at MIC ≥ 8 µg/mL for *Staphylococcus* spp. (22). CLSI M100 breakpoints were used for the comparator agent linezolid. For *S. aureus*, the susceptible and resistant breakpoints were ≤4 and ≥8 µg/mL, respectively. For *S. pneumoniae*, CLSI only provides a susceptible breakpoint, which is ≤2 µg/mL (15).

Quality control (QC) was performed for the testing batch. For linezolid, QC testing followed CLSI recommendations using *S. aureus* ATCC 29213 and *S. pneumoniae* ATCC 49619, with MIC values required to fall within the acceptable ranges specified in CLSI M100 (15). At the time the study was conducted, the CLSI had not established QC MIC ranges for contezolid. The observed contezolid MIC values were 1 µg/mL for *S. aureus* ATCC 29213 (*N* = 1) and 0.5 µg/mL for *S. pneumoniae* ATCC 49619 (*N* = 1). Subsequently, CLSI M100 (36th edition, 2026) established QC ranges of 1–4 µg/mL and 0.5–2 µg/mL for these strains (23).

MIC distributions were summarized using cumulative inhibitory curves defined as the percentage of isolates inhibited at or below each MIC value. Statistical analyses were performed using SPSS version 25.0. Comparisons of MIC distributions between two groups (PNSP vs. PSSP and MSSA vs. MRSA) were performed using the Mann–Whitney *U* test. Comparisons among multiple groups (specimen sources, serotypes, and sequence types) were performed using the Kruskal–Wallis test. A two-sided *P* < 0.05 was considered statistically significant.

## RESULTS

### Clinical isolates

A total of 145 clinical isolates associated with invasive infections were collected. Among these, 64.1% (*n* = 93) were recovered from blood culture specimens, 18.6% (*n* = 27) from CSF, 12.4% (*n* = 18) from puncture fluid (including pleural fluid, peritoneal fluid and joint fluid), 2.1% (*n* = 3) from tissue samples, 1.4% (*n* = 2) from urine, 0.7% (*n* = 1) from bone marrow, and 0.7% (*n* = 1) from catheter tip.

### *In vitro* activity of contezolid against *S. pneumoniae* isolates

**Fig 1:**
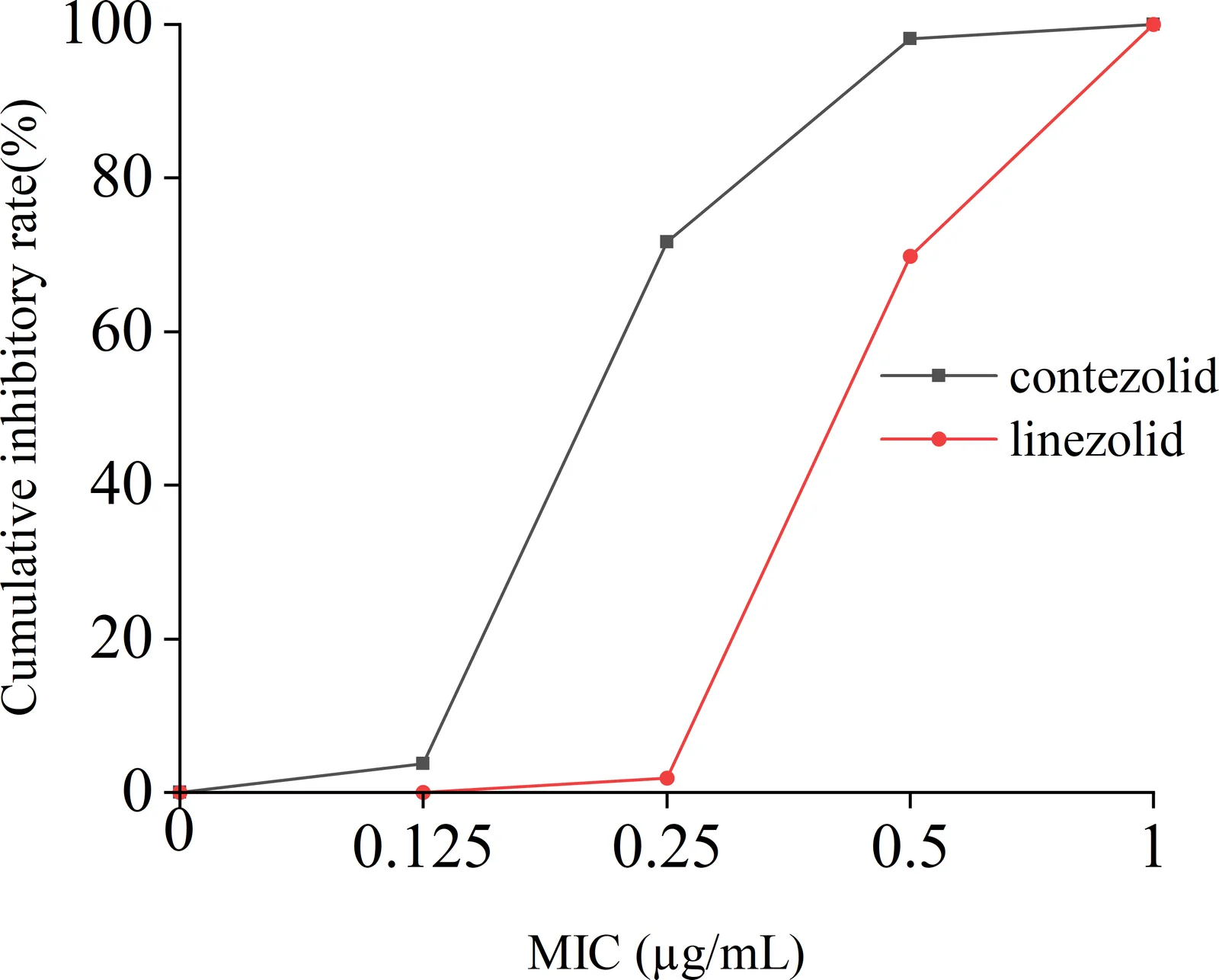
Cumulative inhibitory rate of *S. pneumoniae* (53 isolates) at different MICs.

All *S. pneumoniae* isolates were susceptible to contezolid (Table 1). The MIC values ranged from 0.125 to 1.000 µg/mL, with an MIC_50_ of 0.250 µg/mL and an MIC_90_ of 0.500 µg/mL. In comparison, linezolid exhibited an MIC range of 0.250–1.000 µg/mL, with MIC_50_ and MIC_90_ values approximately twofold higher than those of contezolid. For most isolates, the contezolid MIC was one dilution lower than the corresponding linezolid MIC, indicating superior *in vitro* activity of contezolid. At a concentration of 0.250 µg/mL, the cumulative inhibitory rates were 71.7% for contezolid and 1.9% for linezolid (Fig. 1).

**TABLE 1:** Distribution of MIC values for contezolid and linezolid against test isolates^*a*^^,^^*b*^

Species	Antimicrobial agent	No. of isolates with MIC (µg/mL) of:						MIC (µg/mL)	
		0.125	0.250	0.500	1.000	2.000	4.000	MIC_50_	MIC_90_
*S. pneumoniae* (*n* = 53)	Contezolid	2	36	14	1	0	0	0.250	0.500
	Linezolid	0	1	36	16	0	0	0.500	1.000
PSSP (*n* = 14)	Contezolid	1	11	2	0	0	0	0.250	0.500
	Linezolid	0	1	7	6	0	0	0.500	1.000
PNSP (*n* = 39)	Contezolid	1	25	12	1	0	0	0.250	0.500
	Linezolid	0	0	29	10	0	0	0.500	1.000
*S. aureus* (*n* = 92)	Contezolid	0	1	66	24	1	0	0.500	1.000
	Linezolid	0	0	4	56	31	1	1.000	2.000
MSSA (*n* = 51)	Contezolid	0	0	37	13	1	0	0.500	1.000
	Linezolid	0	0	1	33	17	0	1.000	2.000
MRSA (*n* = 41)	Contezolid	0	1	29	11	0	0	0.500	1.000
	Linezolid	0	0	3	23	14	1	1.000	2.000

^*a*^ PSSP, penicillin-susceptible *Streptococcus pneumoniae*; PNSP, penicillin-nonsusceptible *Streptococcus pneumoniae*; MSSA, methicillin-susceptible *Staphylococcus aureus*; MRSA, methicillin-resistant *Staphylococcus aureus*; MIC, minimum inhibitory concentration.

^*b*^ All MIC values were within the tested concentration range (0.125–4 µg/mL). No isolates exhibited MIC values below 0.125 µg/mL or above 4 µg/mL.

When comparing the MIC distributions of contezolid between PSSP and PNSP isolates, no significant difference in activity was observed (*P* = 0.139). Overall, contezolid maintained potent activity, regardless of the penicillin susceptibility status. Only one PNSP isolate exhibited a higher contezolid MIC of 1.000 µg/mL.

To evaluate the *in vitro* activity of contezolid against *S. pneumoniae* across different specimen sources and serotypes, 53 clinical isolates were included. Serotyping was successful for 52 isolates. Most isolates were obtained from blood and cerebrospinal fluid (Table 2). Contezolid MIC values were predominantly in the range of 0.250–0.500 µg/mL across specimen sources, with no meaningful differences observed between groups (*P* = 0.807).

**TABLE 2:** MIC distribution of contezolid against testisolates from different specimen sources^*a*^

Species	Specimen sources	No. of isolates	MIC distribution (*n*)					
			0.125 µg/mL	0.250 µg/mL	0.500 µg/mL	1.000 µg/mL	2.000 µg/mL	4.000 µg/mL
*S. pneumoniae*	Blood culture	25	1	17	7	0	0	0
	Cerebrospinal fluid	25	1	16	7	1	0	0
	Pleural effusion	2	0	2	0	0	0	0
	Catheterized urine	1	0	1	0	0	0	0
	Total	53	2	36	14	1	0	0
*S. aureus*	Blood culture	68	0	0	46	21	1	0
	Puncture fluid^*b*^	16	0	1	14	1	0	0
	Tissue	3	0	0	3	0	0	0
	Cerebrospinal fluid	2	0	0	1	1	0	0
	Bone marrow	1	0	0	0	1	0	0
	Catheter tip	1	0	0	1	0	0	0
	Midstream urine	1	0	0	1	0	0	0
	Total	92	0	1	66	24	1	0

^*a*^ MIC, minimum inhibitory concentration. The total values correspond to the total numbers of isolates for each specimen source category.

^*b*^ Puncture fluid, including pleural fluid, peritoneal fluid, and joint fluid.

Among the 52 *S. pneumoniae* isolates, serotype 19F was the most common, followed by 6B, 14, 19A, 15A, and 23F. Contezolid exhibited consistently low MIC values across serotypes, with no substantial variation observed (*P* = 0.818). Notably, one CSF-derived serotype 15B PNSP isolate displayed a higher MIC value of 1.000 µg/mL; for this isolate, the MICs of linezolid and penicillin were 1.000 and 2.000 µg/mL, respectively.

### *In vitro* activity of contezolid against *S. aureus* isolates

**Fig 2:**
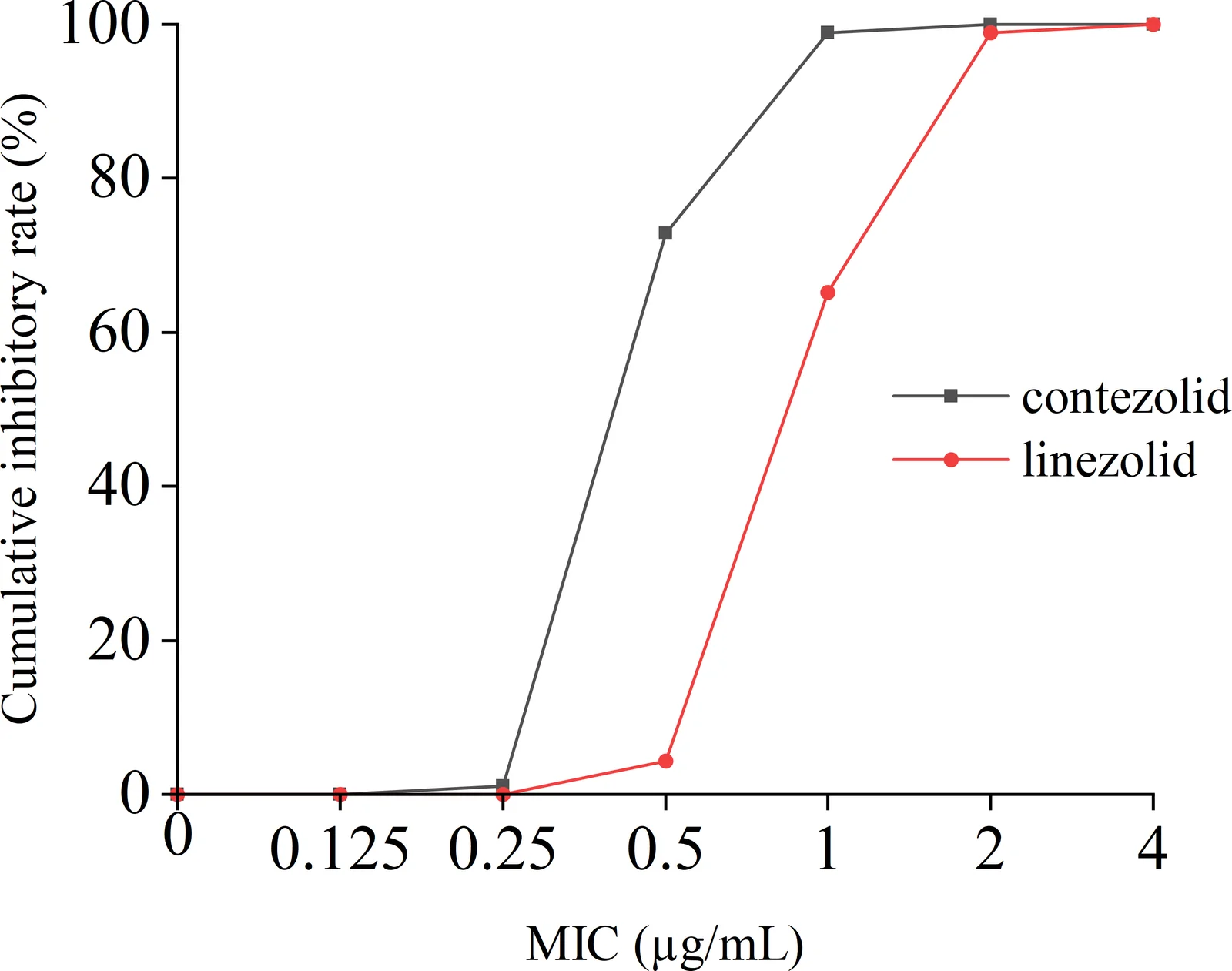
Cumulative inhibitory rate of *S. aureus* (92 isolates) at different MICs.

All 92 *S. aureus* isolates were susceptible to contezolid and linezolid (Table 1). Contezolid exhibited an MIC range of 0.250–2.000 µg/mL, with an MIC_50_ of 0.500 µg/mL and an MIC_90_ of 1.000 µg/mL (Table 1). In comparison, linezolid had an MIC_50_ of 1.000 µg/mL and an MIC_90_ of 2.000 µg/mL. Cumulative inhibition curve analysis (Fig. 2) demonstrated that at a concentration of 0.500 µg/mL, contezolid achieved a cumulative inhibitory rate of 72.8%, markedly higher than that of linezolid (4.4%). Contezolid demonstrated potent *in vitro* activity against both MSSA and MRSA, with comparable MIC distributions between the two groups (*P* = 0.671).

Among the 92 *S. aureus* isolates, most were recovered from blood culture specimens. All isolates were susceptible to contezolid, with MIC values predominantly ≤0.500 µg/mL. A subset of isolates (*n* = 25) exhibited MICs of 1–2 µg/mL, most of which were derived from blood specimens (Table 2).

The predominant clonal lineages were ST22, ST59, and ST398. Contezolid showed similar activity across different sequence types, with no substantial differences in MIC distributions (*P* = 0.339). One ST15 MSSA isolate from blood exhibited the highest contezolid MIC (2.000 µg/mL), with a corresponding linezolid MIC of 2.000 µg/mL, and was considered an outlier.

## DISCUSSION

Contezolid is a novel oxazolidinone antibacterial agent approved in China for the treatment of complicated skin and soft tissue infections and has received Qualified Infectious Disease Product designation from the U.S. Food and Drug Administration for acute bacterial skin and skin structure infections (10). Phase III clinical trials demonstrated non-inferior efficacy to linezolid and an improved safety profile, particularly with respect to hematologic toxicity and monoamine oxidase inhibition (10).

At present, contezolid has no approved indication for pediatric use, and systematic data on its pharmacokinetics (PK), pharmacodynamics (PD), safety, or efficacy in children remain limited (22). Nevertheless, the increasing prevalence of antimicrobial resistance among pediatric gram-positive pathogens highlights the need to explore alternative antimicrobial agents with favorable activity and safety profiles in this vulnerable population.

To our knowledge, this study is the first to comprehensively evaluate the *in vitro* activity of contezolid against a large collection of *S. pneumoniae* and *S. aureus* isolates from pediatric patients with invasive infections. A key finding was that contezolid exhibited potent and consistent *in vitro* activity against all tested isolates, including PNSP and MRSA, providing microbiological evidence to support further investigation in pediatric settings.

For *S. pneumoniae*, contezolid exhibited higher *in vitro* antibacterial activity than linezolid, consistent with findings from adult or mixed-age populations (9, 24). Most isolates were recovered from blood cultures and CSF, and no notable differences in susceptibility were observed by specimen source, suggesting stable activity across invasive infection sites. Contezolid also maintained potent *in vitro* activity across a broad range of pneumococcal serotypes identified in this study. Representative serotypes observed in higher frequency included 19F, 19A, 15A, 6B, and 14, which have also been commonly reported in pediatric pneumococcal populations (25, 26).

The MIC_50_ and MIC_90_ values of contezolid against *S. pneumoniae* in the present study were 0.250 and 0.500 µg/mL, respectively, which are lower than the values of 1.000/1.000 µg/mL reported in adult or mixed-age populations (9). However, our findings are supported by a multicenter study from China, which reported MIC_50/90_ values of 0.500/0.500 µg/mL, closely aligned with our results within one twofold dilution (27). Notably, more than 50% of isolates in that study were derived from pediatric patients, which may partially contribute to the lower MIC values observed. Importantly, the present study and the studies cited in references 9 and 27 used the same reference broth microdilution methodology in accordance with CLSI M07, supporting the comparability of the MIC results across these studies. Overall, these differences are likely multifactorial and may reflect variations in study populations, geographic distribution, and strain composition. Although contezolid is not currently approved for the treatment of respiratory tract infections, oxazolidinones, such as linezolid, are well established for severe pneumonia and other invasive respiratory infections caused by gram-positive pathogens (28). Given the similar mechanism of action and favorable *in vitro* activity observed here, contezolid may represent a potential alternative for invasive pneumococcal infections.

For *S. aureus*, contezolid also demonstrated potent *in vitro* activity, with MIC values lower than those of linezolid for the majority of isolates. This is consistent with Carvalhaes et al. (9), which reported contezolid MIC_50/90_ values of 0.5/1 µg/mL for 606 *S. aureus* isolates from Europe and North America, identical to those observed in the present study. Although no specific analysis of pediatric epidemic clones was performed, the data support stable activity across diverse *S. aureus* populations (9). Notably, contezolid exhibited similar MIC values for both MRSA and MSSA and maintained stable *in vitro* activity against the major pediatric epidemic clones (e.g., ST22 and ST59).

Two isolates with slightly elevated contezolid MIC values within the susceptible range were identified, including one *S*. *pneumoniae* serotype 15B isolate (contezolid MIC, 1 µg/mL; linezolid MIC, 1 µg/mL; penicillin MIC, 2 µg/mL) and one MSSA ST15 isolate (contezolid MIC, 2 µg/mL; linezolid MIC, 2 µg/mL). The MIC values of both isolates were confirmed by repeat broth microdilution testing, yielding consistent results. The elevated contezolid MICs were accompanied by similarly elevated linezolid MICs, which were also in the susceptible range. Although strain-specific factors cannot be excluded, the available data do not permit further conclusions regarding the underlying basis of these findings.

PK/PD data provide additional context regarding the potential clinical relevance of the potent *in vitro* activity observed in this study. After oral administration, contezolid is rapidly absorbed. The bioavailability of contezolid was estimated as 60% with regular diet intake and 100% with high-fat diet (29, 30). In addition, contezolid exhibits wide distribution with high plasma protein binding, and no significant accumulation has been observed with repeated dosing (31, 32). In adult populations, Monte Carlo simulations have indicated that the standard oral contezolid regimen (800 mg every 12 h) achieves a probability of target attainment (PTA) exceeding 90% against MRSA isolates with MICs up to 4.0 µg/mL (33). In the present study, all *S. aureus* isolates had MICs ≤ 1.0 µg/mL, which is below the MIC threshold associated with >90% PTA in these adult PK/PD simulations. However, these PK/PD data were derived from adult populations and should not be directly extrapolated to pediatric patients. Pediatric-specific PK/PD and clinical data for contezolid, particularly for bloodstream and other sterile-site infections, remain limited.

Contezolid exerts its antibacterial effect by specifically binding to the 23S rRNA of the 50S ribosomal subunit of bacteria (34, 35). This unique binding site may reduce the likelihood of cross-resistance with non-oxazolidinone agents (35). Previous studies have demonstrated a low spontaneous mutation frequency (34). In addition, a serial passage experiment further indicates a lower propensity for resistance development compared with linezolid (36). In this context, contezolid may offer potential advantages over linezolid.

Overall, contezolid demonstrated potent and consistent *in vitro* activity against *S. aureus* and *S. pneumoniae*, including PNSP and MRSA. These findings, together with its favorable pharmacological profile, warrant further investigation of contezolid for pediatric invasive infections caused by resistant *S. pneumoniae* and *S. aureus*.

In summary, contezolid exhibited consistently low MIC values against invasive gram-positive bacteria from pediatric patients, with MIC_50_ and MIC_90_ values lower than those of linezolid. As pediatric-specific PK/PD targets remain to be fully established, these results should be interpreted as preliminary *in vitro* evidence supporting further pharmacokinetic and pharmacodynamic studies rather than direct evidence of clinical efficacy. Additional prospective studies are warranted to evaluate the safety and efficacy of contezolid in children and clarify its potential role in clinical practice.

## Data Availability

The data supporting the findings of this study are available from the corresponding author upon reasonable request.
